# The Cyanobacterial Role in the Resistance of Feather Mosses to Decomposition—Toward a New Hypothesis

**DOI:** 10.1371/journal.pone.0062058

**Published:** 2013-04-15

**Authors:** Kathrin Rousk, Thomas H. DeLuca, Johannes Rousk

**Affiliations:** 1 School of the Environment, Natural Resources, & Geography, Bangor University, Bangor, Gwynedd, United Kingdom; 2 School of Environment and Forest Sciences, University of Washington, Seattle, Washington, United States of America; 3 Microbial Ecology, Lund University, Lund, Sweden; DOE Pacific Northwest National Laboratory, United States of America

## Abstract

Cyanobacteria-plant symbioses play an important role in many ecosystems due to the fixation of atmospheric nitrogen (N) by the cyanobacterial symbiont. The ubiquitous feather moss *Pleurozium schreberi* (Brid.) Mitt. is colonized by cyanobacteria in boreal systems with low N deposition. Here, cyanobacteria fix substantial amounts of N_2_ and represent a potential N source. The feather moss appears to be resistant to decomposition, which could be partly a result of toxins produced by cyanobacteria. To assess how cyanobacteria modulated the toxicity of moss, we measured inhibition of bacterial growth. Moss with varying numbers of cyanobacteria was added to soil bacteria to test the inhibition of their growth using the thymidine incorporation technique. Moss could universally inhibit bacterial growth, but moss toxicity did not increase with N_2_ fixation rates (numbers of cyanobacteria). Instead, we see evidence for a negative relationship between moss toxicity to bacteria and N_2_ fixation, which could be related to the ecological mechanisms that govern the cyanobacteria – moss relationship. We conclude that cyanobacteria associated with moss do not contribute to the resistance to decomposition of moss, and from our results emerges the question as to what type of relationship the moss and cyanobacteria share.

## Introduction

Symbiotic relationships between plants and microbes have been proposed to be important drivers of evolution [1]. Cyanobacteria are widespread and well-known symbionts, colonizing bryophytes, lichens and higher plants [2], [3], [4]. Cyanobacteria are facultative autotrophs, generally fixing atmospheric carbon (C) and nitrogen (N), but are also known to use host-C through symbiotic relationships [4]. The general assumption is that cyanobacteria-plant associations are mutualistic symbioses: the plant host receives N in the form of ammonium (NH_4_^+^) or amino acids and in return provides carbohydrates, shelter and protection [5], [6], suggesting ecological specialization where the cyanobacteria down-regulate their own photosynthesis [3], [7].

In the northern boreal forest, atmospheric N deposition is generally low (1–3 kg N ha^−1^ yr^−1^), however, mosses colonized by N_2_ fixing cyanobacteria may contribute a further 2 kg N ha^−1^ yr^−1^, thus representing a major N input pathway in this pristine environment [8], [9]. Due to the strong N limitation of these ecosystems [10], the moss-cyanobacteria association characterizes the productivity of the ecosystem as well as its biogeochemical budget. One example is the relationship between one of the dominant primary producers in mid- to late succession boreal forests [8], the feather moss *Pleurozium schreberi* (Brid.) Mitt., and associated cyanobacteria. Substantial amounts of atmospheric N_2_ are fixed by cyanobacteria colonizing *P. schreberi* [8]. However, this process is sensitive to N inputs: higher N input through deposition results in lower numbers of cyanobacteria in the feather mosses and, consequently, in lower N_2_ fixation rates [9], [11], [12].

In spite of their dominant presence as a ground cover in the boreal forest ([8], [12], feather mosses are consumed by very few herbivores [13]. Although other bryophytes have been found to be even more resistant to decomposition [14], [15], it has been noted that even the herbivores that graze mosses appear to specifically avoid the ubiquitous *P. schreberi* and *Hylocomium splendens* [16]. Additionally, moss litter is known to be highly resistant to microbial decomposition in high latitude ecosystems [17]. However, the low quality of moss as a substrate or food source [13], [17] appears to be an insufficient explanation for the lack of utilization of this ample potential resource. The cyanobacteria that associate with feather mosses are known to produce toxins [3], [18], [19], which thus could play an important role in the resistance of mosses to grazing and decomposition. This would be a hitherto unrecognized dimension of the relations between feather moss and their cyanobacterial colonizers.

In this experiment we assess the contribution by cyanobacteria to the resistance to decomposition characteristic for mosses. More precisely, we hypothesize that moss toxicity increases with higher levels of cyanobacterial colonization and activity (N_2_ fixation). To assess this hypothesis, we used a microbial bioassay to estimate the toxicity of *P. schreberi* by determining its propensity to inhibit soil bacterial growth [20], [21], [22] across a gradient of cyanobacterial colonization in similar mid-later succession boreal forests in northern Sweden.

## Materials and Methods

### Sampling

We used shoots of the feather moss *P. schreberi* from six independent sites (all>1 km apart) distributed in four different mid- to late succession forests (Dötternoive, Borup, 2 sites from Nyvall, 2 sites in Kuottavare, more than 200 m apart; permission to sample granted by Norrbotten County Administrative board, Sweden, to Prof. M.C. Nilsson, Swedish Agricultural University, Umeå, Sweden) in Northern Sweden that have been shown to encompass a wide range of different N_2_ fixation rates needed for our experiment [12]. The sites were chosen to be as similar as possible in other aspects, and include a tree community of similar age (150–300 years; see [8], [12], [23]), successional stage, and nutrient status. The sites were located between latitude 64–65°N and longitude 18–19°E and have been described previously [8], [12], [23] (see also Table 1). Mean annual temperature and precipitation were 1 °C and 570 mm, respectively. The vegetation consists of *Pinus sylvestri*s; *Picea abies*; *Vaccinium vitis-idaea*; *V. myrtillus*; *Empetrum hermaphroditum*; *H. splendens* and *P. schreberi*. Soils at the sites are classified as Typic Haplocryods (FAO, Cambic Podzol). While we are not able to exclude the possibility that other factors in addition to cyanobacterial colonization and N_2_ fixation activity were different among the six studied sites, it is not evident that any such factors could also have directly affected the mosses propensity to inhibit bacterial growth.

**Table 1:** Mean values (N = 18)±SE of the main soil properties from the moss- sampling sites in Northern Sweden.

pH	TC	TN	C/N	DOC	DON	DIN
4.4±0.3	369±23	11±3.6	48±1	1.46±0.17	0.02±0.02	0.002±0.0002

Data for soil nutrients are given in mg g^−1^ soil dry weight.

TC = Total Carbon; TN = Total Nitrogen; DOC = Dissolved Organic Carbon; DON = Dissolved Organic Nitrogen; DIN = Dissolved Inorganic Nitrogen. C/N is the ratio between TC and TN.

### N_2_ fixation

N_2_ fixation was measured using a ^15^N calibrated acetylene reduction assay [24] as previously described [23]. Briefly, 10 moss shoots(>800 leaves shoot^−1^) from each site were placed in a 20 ml tube, sealed and 10% of the headspace was replaced with acetylene. Moss samples were incubated for 24 h at room temperature. Ethylene generated in the headspace by the cyanobacterial nitrogenase enzyme was measured by gas chromatography equipped with a flame ionization detector (Varian, Santa Clara, USA). The fresh moss shoots were ground using a ball mill (2 min., 30 s^−1^) forming a moss slurry, which was subsequently used in the bacterial growth assay to determine toxicity (below). Until processing, samples were kept at 4°C.

### Cyanobacterial counts

Five different fronds with 10 leaves each were counted per sample, and moss leaves were destructively harvested from the fronds through scraping to enable microscopy counting. Cyanobacteria cells were then counted using an ultraviolet-fluorescence micrograph (Zeiss Axiophot 2 fluorescence microscope with 20× magnification) with a green filter. Cyanobacteria possess auto-fluorescent phycobiliproteins to capture light; therefore no staining is needed to visualize them using a fluorescence microscope.

### Soil bacterial growth and toxicity assay

Soil bacterial growth rates were estimated using the thymidine incorporation technique [25], [26]. The reason to use the thymidine incorporation technique as opposed to leucine incorporation technique was that the latter is susceptible to confounding isotope dilution effects in plant tissue samples, which the present assay would be sensitive to. I.e., non-radioactive leucine concentrations were likely to scale with the moss-addition, causing unwanted isotope dilution effects. The determination of moss toxicity was otherwise determined analogously to salt toxicity in [21]. Briefly, 2 g of wet weight (ww) of soil (a composite and homogenized sample of the soil from all sites) was mixed with 20 ml of distilled water. The soil suspensions were vortexed for 3 min and then centrifuged for 10 min at 1000 *g* to obtain a bacterial suspension in the supernatant. From the soil bacterial suspension, 1.35 ml was used for the thymidine incorporation measurements in 2 ml microcentrifuge tubes. Subsequently, 0.15 ml moss (see below) was added to the 1.35 ml of the bacterial suspension in the microcentrifuge tubes.

Eight different concentrations of moss was used for each sample to determine its toxicity to bacterial growth; 0, 0.14, 0.41, 1.2, 3.7, 11, 33, 100 mg moss ml^−1^ were used in the moss toxicity determination as final concentrations in the bacterial suspensions. Distilled water was added to the control and to dilute the moss in the dilution series. Thymidine (TdR; [*Methyl*-^3^H]thymidine, 25 Ci mmol^−1^, 1.0 mCi ml^−1^, Perkin Elmer) was added to a final concentration of 130 nM in the bacterial suspensions. The samples were then incubated at approximately 20 °C for 2 h. The incorporation of TdR was terminated by the addition of 75 µL of 100% trichloroacetic acid. Non-incorporated TdR was then removed as described in [26], and TdR incorporation was measured in a scintillation counter (Wallac EG&G, Milton Keynes, UK).

### Chemical analyses

We also investigated the N concentration in moss tissue of samples collected along gradients of cyanobacterial N_2_ fixation in *Pleurozium schreberi* induced by road pollution in boreal forests (for N_2_ fixation data and additional details, see [12]). Moss samples were dried at 80 °C for 24 h and ground to a fine powder using a ball mill. Total N (TN) in moss samples were analysed by oxidative combustion using an elemental analyzer interfaced to a continuous flow isotope ratio mass spectrometer (IRMS) (Sercon Ltd., Cheshire, UK).

Total phenols were measured in moss tissue that was first ground by placing 1 g dry weight equivalent of fresh moss tissue in a ball mill and then suspended in 10 ml of distilled water. Samples were centrifuged at 4000 rpm and the supernatant analyzed for phenols by using the Folin and Ciocalteau's reagent [27] and absorbance measured at 725 nm using a microplate reader.

### Statistical analyses and calculations

Toxicity values were expressed as the concentration resulting in 50% inhibition (EC_50_)) of the bacterial growth in the soil suspensions. This is a universally adopted concept to toxicology and central to all ecotoxicology (e.g. [20], [21], [22]). More toxic moss inhibited the bacterial growth at lower concentrations and, therefore, has a lower value of EC_50_ than a less toxic moss. The EC_50_-values of the bacterial communities were calculated using a logistic model, *Y* = *c*/[1+e*^b^* ^(*X−a*)^], where *Y* is the TdR incorporation rate, *X* is the logarithm of the moss concentration, *a* is the value of log(EC_50_), *c* is the TdR incorporation rate in the control, and *b* is a parameter (the slope) indicating the inhibition rate. Kaleidagraph 4.0 for Mac (Synergy software) was used to fit a logistic curve to the data using the equation. Relationships between variables of the different sites were investigated using regression analyses or using analysis of variance (ANOVA) with Tukey's HSD post hoc pair-wise comparisons using JMP 9.0 for Mac (SAS Institute, Cary, NC, USA).

## Results

### Toxicity

**Figure 1:**
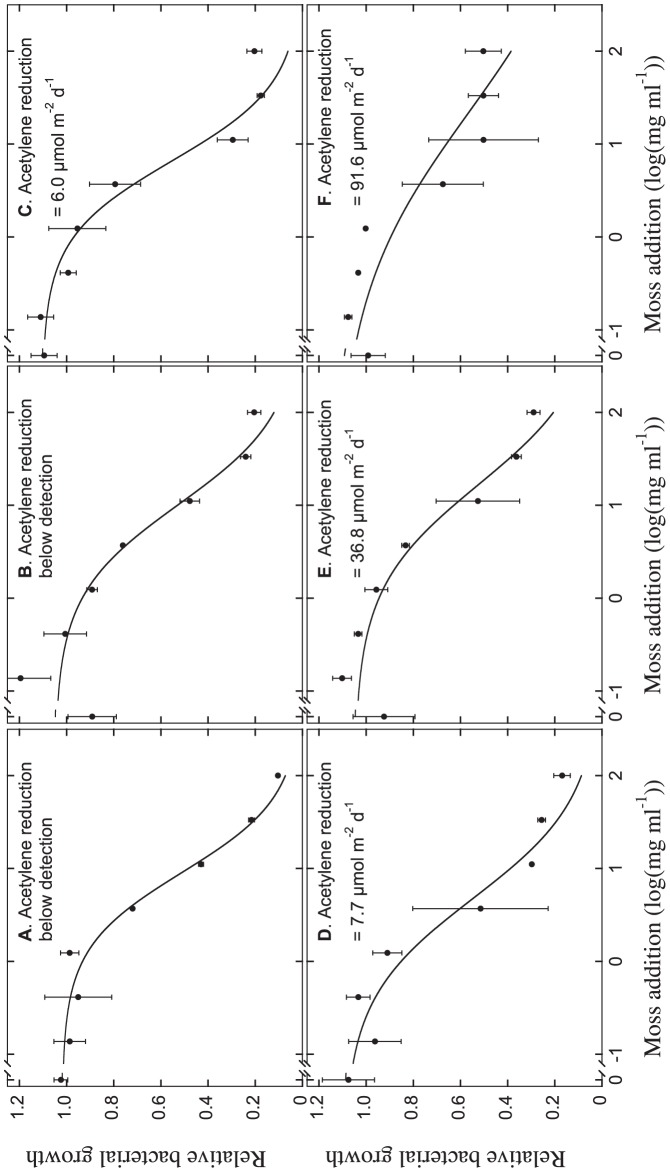
Inhibition of bacterial growth by differently concentrated *Pleurozium schreberi* solutions hosting different numbers of cyanobacteria and with that, different N_2_ fixation rates (A–F). These dose-response relationships were used to establish indices for toxicity (EC_50_ values) that were used in subsequent analyses (Fig. 2). Data points are mean values±1 SE of two determinations, and the fitted logistic curves (see Material and Methods) are based on the eight moss-concentration levels (*n* = 8).

We were able to demonstrate clear dose-response relationships between bacterial growth and moss addition for the six sites studied here (Fig. 1), with lower bacterial growth following exposure to higher concentrations of moss. The relationship between bacterial growth and moss exposure could be modeled well using a logistic model (*R^2^* = 0.99, 0.93, 0.96, 0.96, 0.95 and 0.84 for the six sites, ordered from low to high N_2_ fixation rates/cyanobacteria numbers). Higher concentrations of moss effectively inhibited bacterial growth as indicated by suppression of growth by 90% at the highest moss addition rate.

**Figure 2:**
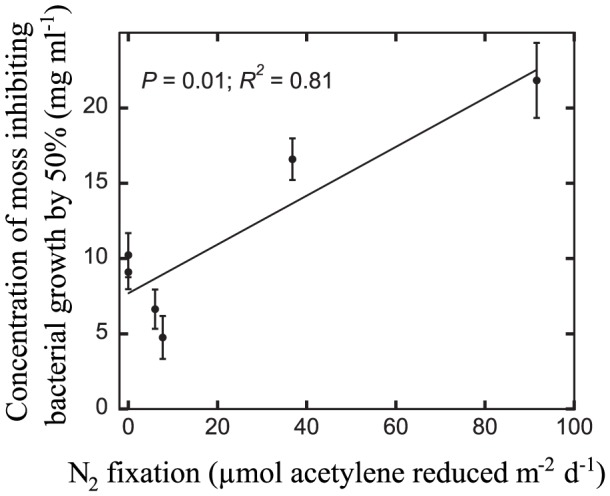
Relation between EC_50_ values as an index for moss toxicity and N_2_ fixation rates in *P. schreberi*. Lower EC_50_ values indicate higher toxicity, and *vice versa*. Shown are mean values ±SE derived from the inhibition curves (Fig. 1).

The EC_50_-values were used as indices for moss toxicity to bacterial growth. The EC_50_-values were positively related to the presence (cyanobacterial cell count, see below; P<0.05; R^2^ = 0.72) and activity (acetylene reduction, see below; P = 0.01; R^2^ = 0.81; Fig. 2) of cyanobacteria on the mosses. This means that moss-toxicity decreased with higher cyanobacterial numbers and N_2_ fixation activity.

### N_2_ fixation and cyanobacterial count

**Figure 3:**
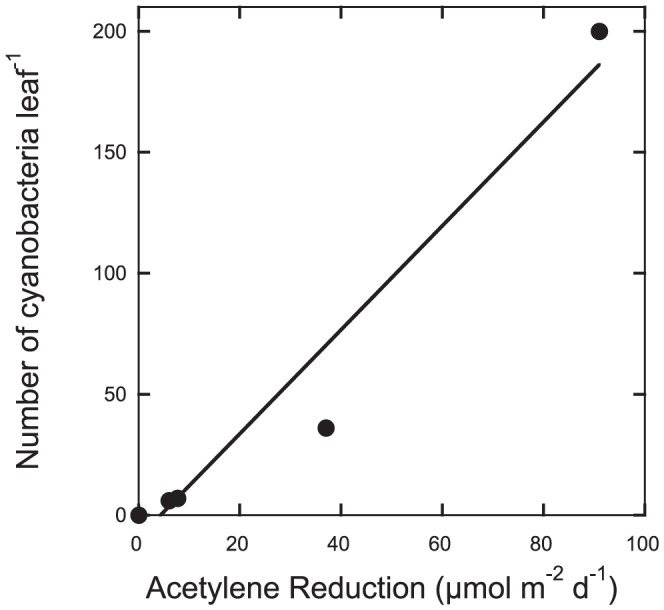
Numbers of cyanobacteria cells leaf**^−1^** in relation to acetylene reduction (**µ**mol m**^−2^** d**^−1^**) in the feather moss *P. schreberi*.

N_2_ fixation rates were found to scale linearly with the numbers of cyanobacteria in the mosses, ranging between 0 and 200 cells leaf^−1^ (P<0.0001; R^2^ = 0.94) (Fig. 3), where low counts corresponded to low rates, and *vice versa*, and where a N_2_ fixation rate of 0 µmol m^−2^ d^−1^ acetylene reduced corresponded to 0 cyanobacteria cells, and a N_2_ fixation rate of 91 µmol m^−2^ d^−1^ acetylene reduced corresponded to 200 cyanobacteria cells leaf^−1^ (16*10^4^ cells shoot^−1^).

### Chemical analyses

Total phenols in moss tissue ranged between 5.4 and 9.0 mg phenol g^−1^ fresh weight (Table 2). No correlation was found between phenols and N_2_ fixation and cyanobacterial numbers or between phenols and EC_50_-values.

**Table 2:** Moss characteristics of *Pleurozium schreberi*-tissue collected in Northern Sweden.

	pH		C/N*		Phenols (mg g^−1^ fw)		Chlorophyll (SPAD units)*	
Site	*mean*	*se*	*mean*	*se*	*mean*	*se*	*mean*	*se*
**A**	4.6	0.12	44	0.5	7	0.2	4.7	0.84
**B**	4.8	0.12	44	0.5	5.4	0.37	4.7	0.84
**C**	4.4	0.13	32	0.8	5.7	0.38	2.5	0.53
**D**	4.4	0.13	32	0.8	9	0.49	2.5	0.53
**E**	4.4	0.13	40	1	6.8	0.19	3.4	1.3
**F**	4.3	0.15	53	0.9	8.4	0.56	3.5	0.81

Values are means±1 SE of 3–6 determinations. * C/N and Chlorophyll concentrations were determined on the same samples for sites A and B, and C and D.

Across well-replicated (3 independent sites, 3 independent replicates in each) and steep N_2_ fixation gradients from 30±9 to 250±40 µmol acetylene reduced m^−2^ d^−1^ (reported in [12]) we saw no differences in moss N-tissue concentrations, which were 35±3.2 and 37±1.9 µg N g^−1^ moss across the same gradient. This showed that the moss-additions in our bacterial toxicity assay that differed in cyanobacterial colonization and N_2_-fixation rate were not confounded by variation in added N in our bacterial assay.

## Discussion

The use of biomarkers has been an invaluable tool to directly assess the toxic effect of substances in the environment [28]. While the interpretation of toxicity assessments based on biomarkers must consider the particular sensitivity or tolerance of the organisms used, it is generally held that the small size of microorganisms (and high area per volume) make them particularly exposed to toxins and thus sensitive indicators of the substances' general toxicity [29]. Moreover, while single species tests can be strongly biased by the properties of the particular strains included and must be supplemented by a battery of tests of different organisms before their effects can be generalized, assessing whole bacterial communities (as in the present study) are more robust in principle: it takes advantage of the naturally highly diverse bacterial communities to provide a continuous toxicity response that is a powerful tool to accurately establish the general propensity of a substance to be toxic [30]. Indeed, previous comparative assessments have validated the use of bacterial growth to assess environmental toxicity (e.g. [31], [32], [33]).

Bacterial growth was universally inhibited by moss additions irrespective of cyanobacterial presence or activity, as shown by the clear dose-response relationship that could be established for all tested mosses. This validated that we could assess the toxicity of moss. We hypothesized that moss toxicity, as indicated by the propensity of mosses to inhibit bacterial growth, would increase with higher numbers of cyanobacteria present. What we found was that cyanobacteria did not contribute at all to the toxicity of moss to bacteria. We cannot rule out a higher susceptibility of the other major decomposer group, fungi, to the toxic potential of mosses. However, in a thorough assessment of the abundance of fungi (ergosterol concentration) in relation to a previously reported N_2_-fixation gradient across fire chronosequence in boreal forests in Northern Sweden (see [23]), we found no differences in fungal presence (J. Rousk & K. Rousk, unpublished). It might be reasonable to suppose that bacteria should be more susceptible than fungi to toxins based on their higher area per volume. However, the fungal role in the decomposition of mosses, and the fungal susceptibility to moss toxicity, still remains to be studied.

That moss can inhibit bacterial growth is not surprising, and probably is partly related to the inhibitory nature of constitutional plant compounds, such as phenols, which have a known inhibitory effect on microorganisms (e.g. [34]). Further, it is possible that phenol content could have been instrumental for much of the toxicity that moss exerted on bacteria in general, since moss from all six sites could effectively suppress bacterial growth at high concentrations (Fig. 1). However, moss phenol content could not be used to explain *differences* in the inhibition of bacterial growth with regard to presence of cyanobacteria, as phenol concentrations in moss samples did not correlate with cyanobacterial colonization or with moss toxicity (EC_50_ values). Moreover, toxins produced by cyanobacteria are not exclusively phenolic, rather the majority are alkaloids and cyclic peptides [18]. Addition of N can result in an inhibition of microbial activity [35], making the tissue concentration of N a putative confounding factor in our analysis. However, we find no differences in the N content of mosses with high compared to low N_2_ fixation rate, effectively falsifying this as an alternative explanation for the inhibition of bacterial growth.

The obtained results on the variation of moss toxicity are of a negative nature, lending no support to our hypothesis stating that cyanobacteria contribute to the toxicity of moss. However, rather than no relationship (i.e. no slope in Fig. 2), we have a suggestion for a higher toxic effect by mosses that was correlated with lower cyanobacteria numbers and activity. Could these results be used to guide our understanding of the hitherto elusive ecology of moss-cyanobacteria relations? A range of observations from previous experiments and field investigations could be combined with our here reported results to develop a way forward to address this.

*P. schreberi* collected in Wales (UK), exposed to relatively high rates of N deposition(>12 kg N ha^−1^ yr^−1^, [36]), are not colonized by cyanobacteria and with that, do not fix N_2_ (K. Rousk & T.H. DeLuca, unpublished). When stored in a laboratory this remained true, but when N starvation was experimentally induced (rinsing with de-ionized water) cyanobacterial colonization and N_2_ fixation both commenced. It has also been found that cutting off the rhizoids and dead moss associated with moss carpets (i.e. severing its N supply from the soil) and returning it to the forest floor leads to a significant increase in cyanobacterial colonizers and N_2_ fixation rates (T.H. DeLuca, unpublished results). Further, the cutting of moss carpets from N rich environments and subsequent transplantation to N poor environments results in colonization of moss leaves by cyanobacteria and increased N_2_ fixation [37]. Taken together, these and other observations suggest that a regulation of cyanobacterial colonization of moss is very sensitive to the environmental growth conditions of the moss.

A possible mechanism for the regulation of cyanobacterial colonization of moss is the production of toxic secondary metabolites [19], such as oxylipins, derived from fatty acids [38], [39]. It is possible that such compounds could be used to control the colonization by bacteria generally, including that of cyanobacteria. The underlying ecology and causality of such a regulation would be ambiguous, however. One possibility would be that moss actively down-regulates the production of toxins during times of N deficiency to enable colonization by cyanobacteria and with that, a cyanobacteria powered endogenous supply of the limiting resource. Another possibility would be that conditions of N deficiency compromised the moss' ability to defend against opportunistic microorganisms generally, and cyanobacteria with their independent N supply in particular. Both these scenarios would imply that we should expect an emerging pattern that should be validated with observation: Moss decomposition should be slower in environments with high ambient N input, where cyanobacterial colonization is lower. Unfortunately we are not aware of a comprehensive data-set where this prediction could be evaluated, emphasizing a knowledge gap that urgently needs to be filled. These forwarded hypotheses call into question the placement of the cyanobacterial-moss symbiosis on the mutualism – parasitism continuum, which presently is an active area of research [40], [41]. The substantial work on the mycorrhizal-plant symbiosis [42] could act as a lens through which to focus experimental work to resolve the ecological interactions between the associated moss and cyanobacteria.

To conclude, we find no support for any contribution by cyanobacteria to the ability of feather mosses to resist decomposition. Instead, our results suggest a negative relationship between moss toxicity and cyanobacterial colonization. Our findings generate novel questions regarding the type of relationship that characterizes the ecology of moss and cyanobacteria – mutualistic or parasitic symbiosis?

## References

[1] Wernegreen JJ (2004) Endosymbiosis: Lessons in Conflict Resolution. PLoS Biol 2: 308–311. 10.1371/journal.pbio.0020068 PMC368163 15024418

[2] Rai NA, Söderbäck E, Bergman B (2000) Cyanobacterium-plant symbioses. New Phytol 147: 449–481. 10.1046/j.1469-8137.2000.00720.x 33862930

[3] Adams DG, Duggan PS (2008) Cyanobacteria-bryophyte symbioses. J Exp Bot 59: 1047–1058. 18267939 10.1093/jxb/ern005

[4] Meeks JC (2009) Physiological adaptations in nitrogen-fixing Nostoc-plant symbiotic associations. Microb Monogr 8: 181–205.

[5] Meeks JC, Enderlin CS, Joseph CM, Chapman JS, Lollar MWL (1985) Fixation of N_2_ and transfer of fixed nitrogen in the *Anthoceros-Nostoc* symbiotic association. Planta 164: 406–414. 24249612 10.1007/BF00402954

[6] Steinberg NA, Meeks JC (1991) Physiological sources of reductant for nitrogen fixation activity in Nostoc sp. strain UCD 7801 in symbiotic association with *Anthoceros punctatus* . J Bacteriol 173: 7324–7329. 1938924 10.1128/jb.173.22.7324-7329.1991 PMC209240

[7] Meeks JC, Elhai J (2002) Regulation of cellular differentiation in filamentous cyanobacteria in free-living and plant-associated symbiotic growth states. Microbiol Molecul Biol Rev 66: 94–121. 10.1128/MMBR.66.1.94-121.2002 PMC120779 11875129

[8] DeLuca TH, Zackrisson O, Nilsson MC, Sellstedt A (2002) Quantifying nitrogen-fixation in feather moss carpets of boreal forests. Nature 419: 917–920. 12410308 10.1038/nature01051

[9] Gundale MJ, DeLuca TH, Nordin A (2011) Bryophytes attenuate anthropogenic nitrogen inputs in boreal forests. Glob Ch Biol 17: 2743–2753.

[10] Tamm CO (1991) Nitrogen in terrestrial ecosystems. Berlin: Springer.

[11] DeLuca TH, Zackrisson O, Gundale MJ, Nilsson MC (2008) Ecosystem feedbacks and nitrogen fixation in boreal forests. Science 320: 1181–1181. 18511682 10.1126/science.1154836

[12] Ackermann K, Zackrisson O, Rousk J, Jones DL, DeLuca TH (2012) N_2_ fixation in feather mosses is a sensitive indicator of N deposition in boreal forests. Ecosystems 15: 986–998.

[13] Prins HHT (1982) Why Are Mosses Eaten in Cold Environments Only? Oikos 38: 374–380.

[14] Lang SI, Cornelissen JHC, Klahn T, van Logtestijn RSP, Schweikert W, et al (2009) An experimental comparison of chemical traits and litter decomposition rates in a diverse range of subarctic bryophytes. J Ecology 97: 886–900.

[15] Fenton NJ, Bergeron Y, Paré D (2010) Decomposition rates of bryophytes in managed boreal forests: influence of bryophyte species and forest harvesting. Plant Soil 336: 499–508.

[16] Eskelinen O (2002) Diet of the wood lemming *Myopus schisticolor* . Annal Zoologici Fennici 39: 49–57.

[17] Hobbie SE (1996) Temperature and Plant Species Control Over Litter Decomposition in Alaskan Tundra. Ecolog Monographs 66: 503–522.

[18] Cox PA, Banack SA, Murch SJ, Rasmussen U, Tien G, et al (2005) Diverse taxa of cyanobacteria produce β-*N*-methylamino-l-alanine, a neurotoxic amino acid. Proc Nat Acad Sci USA 102: 5074–5078. 15809446 10.1073/pnas.0501526102 PMC555964

[19] Kaasalainen U, Fewer DP, Jokela J, Wahlsten M, Sivonen K, et al (2012) Cyanobacteria produce a high variety of hepatotoxic peptides in lichen symbiosis. Proc Nat Acad Sci USA 109: 5886–5891. 22451908 10.1073/pnas.1200279109 PMC3326460

[20] Aldén-Demoling L, Bååth E (2008) The use of leucine incorporation to determine the toxicity of phenols to bacterial communities extracted from soil. Appl Soil Ecol 38: 34–41.

[21] Rousk J, Elyaagubi FK, Jones DL, Godbold DL (2011) Bacterial salt tolerance is unrelated to soil salinity across an arid agroecosystem salinity gradient. Soil Biol Biochem 43: 1881–1887.

[22] Rousk J, Ackermann K, Curling SF, Jones DL (2012) Comparative toxicity of nanoparticulate CuO and ZnO to soil bacterial communities. PLoS ONE 7: e34197 doi:10.1371/journal.pone.0034197. 22479561 10.1371/journal.pone.0034197 PMC3315546

[23] Zackrisson O, DeLuca TH, Nilsson MC (2004) Nitrogen fixation increases with successional age in boreal forests. Ecology 85: 3327–3334.

[24] Schöllhorn R, Burris RH (1967) Acetylene as a competitive inhibitor of nitrogen fixation. Proc Nat Acad Sci 58: 213–218. 5231601 10.1073/pnas.58.1.213 PMC335619

[25] Bååth E (1992) Thymidine incorporation into macromolecules of bacteria extracted from soil by homogenization–centrifugation. Soil Biol Biochem 24: 1157–1165.

[26] Bååth E, Pettersson M, Söderberg KH (2001) Adaptation of a rapid and economical microcentrifugation method to measure thymidine and leucine incorporation by soil bacteria. Soil Biol Biochem 33: 1571–1574.

[27] Swain T, Hillis WE (1959) The phenolic constituents of *Prunus domestica*. I. The quantitative analysis of phenolic constituents. J Agri Food Sci 10: 63–68.

[28] Van Gestel CAM, van Brummelen TC (1996) Incorporation of the biomarkers concept in ecotoxicology calls for a redefinition of terms. Ecotoxicol 5: 217–225. 10.1007/BF00118992 24193812

[29] Kahru A, Dubourguier HC, Blinova I, Ivask A, Kasemets K (2008) Biotests and Biosensors for Ecotoxicology of Metal Oxide Nanoparticles: A Minireview. Sensors 8: 5153–5170. 27873807 10.3390/s8085153 PMC3705494

[30] Brandt KK, Jorgensen NOG, Nielsen TH, Winding A (2004) Microbial community-level toxicity testing of linear alkylbenzene sulfonates in aquatic microcosms. FEMS Microbiol Ecol 49: 229–241. 19712417 10.1016/j.femsec.2004.03.006

[31] Ore S, Mertens J, Brandt KK, Smolders E (2010) Copper toxicity to bioluminescent Nitrosomanas europaea in soil is explained by the free metal ion activity in pore water. Environ Sci Technol 44: 9201–9206. 21047118 10.1021/es1026294

[32] Brandt KK, Sjöholm OR, Krogh KA, Halling-Sorensen B, Nybroe O (2009) Increased pollution-induced bacterial community tolerance to sulfadiazine in soil hotspots amended with artificial root exudates. Environ Sci Technol 43: 2963–2968. 19475978 10.1021/es803546y

[33] Aldén-Demoling L, Bååth E, Greve G, Wouterse M, Schmitt H (2009) Effects of sulfamethoxazole on soil microbial communities after adding substrate. Soil Biol Biochem 41: 840–848.

[34] Vanderpoorten A, Goffinet B (2009) Introduction to Bryophytes. Cambridge: Cambridge University Press.

[35] Aldén L, Demoling F, Bååth E (2001) Rapid method of determining factors limiting bacterial growth in soil. Appl Environ Microbiol 67: 1830–1838. 11282640 10.1128/AEM.67.4.1830-1838.2001 PMC92804

[36] Jones MLM, Wallace HL, Norris D, Brittain SA, Haria S, et al (2004) Changes in Vegetation and soil characteristics in coastal sand dunes along a gradient of atmospheric nitrogen deposition. Plant Biol 6: 598–605. 15375731 10.1055/s-2004-821004

[37] DeLuca TH, Zackrisson O, Gentili F, Sellstedt A, Nilsson MC (2007) Ecosystem controls on nitrogen fixation in boreal feather moss communities. Oecologia 152: 121–130. 17219131 10.1007/s00442-006-0626-6

[38] Croisier E, Rempt M, Pohnert G (2010) Survey of volatile oxylipins and their biosynthetic precursors in bryophytes. Phytochem 71: 574–580. 10.1016/j.phytochem.2009.12.004 20079505

[39] Matsui K (2006) Green leaf volatiles: hydroperoxide lyase pathway of oxylipin metabolism. Curr Opin Plant Biol 9: 274–280. 16595187 10.1016/j.pbi.2006.03.002

[40] Kiers ET, Denison RF (2008) Sanctions, cooperation, and the stability of plant-rhizosphere mutualisms. Ann Rev Ecol Evol Syst 39: 215–236.

[41] Grman E (2012) Plant species differ in their ability to reduce allocation to non-beneficial arbuscular mycorrhizal fungi. Ecology 93: 711–718. 22690621 10.1890/11-1358.1

[42] Smith SE, Read DJ (2008) Mycorrhizal Symbiosis., Third Edition, Academic Press.

